# Disease Pathway Cut for Multi-Target drugs

**DOI:** 10.1186/s12859-019-2638-3

**Published:** 2019-02-13

**Authors:** Sunjoo Bang, Sangjoon Son, Sooyoung Kim, Hyunjung Shin

**Affiliations:** 1 Department of Industrial Engineering, Ajou University, 206, World cup-ro, Yeongtong-gu, Suwon-si, Gyeonggi-do 16499 Republic of Korea; 2 Department of Psychiatry, Ajou University School of Medicine, 206, World cup-ro, Yeongtong-gu, Suwon-si, Gyeonggi-do 16499 Republic of Korea; 3 Department of Surgery, Thyroid Cancer Center, Gangnam Severance Hospital, Institute of Refractory Thyroid Cancer, Yonsei University College of Medicine, 211, Eonju-ro, Gangnam-gu, Seoul, Republic of Korea

**Keywords:** Target gene identification, Disease pathway, Directed PPI, Pathway network, Min-cut algorithm

## Abstract

**Background:**

Biomarker discovery studies have been moving the focus from a single target gene to a set of target genes. However, the number of target genes in a drug should be minimum to avoid drug side-effect or toxicity. But still, the set of target genes should effectively block all possible paths of disease progression.

**Methods:**

In this article, we propose a network based computational analysis for target gene identification for multi-target drugs. The min-cut algorithm is employed to cut all the paths from onset genes to apoptotic genes on a disease pathway. If the pathway network is completely disconnected, development of disease will not further go on. The genes corresponding to the end points of the cutting edges are identified as candidate target genes for a multi-target drug.

**Results and conclusions:**

The proposed method was applied to 10 disease pathways. In total, thirty candidate genes were suggested. The result was validated with gene set enrichment analysis software, PubMed literature review and *de facto* drug targets.

## Background

Studies on biomarker discovery have been moving the focus from single genes to multiple genes that interact in a cell [1–4]. The recent drug development researches are underway in this trend, because the single target approach may remain a certain possibility of disease progression since it may be developed along the other paths. On the other hands, the multiple target approach is expected to be more effective by simultaneously blocking multiple paths of disease progression. However, it is reckless to consider all possible combinations of genes since it may be not only computationally intractable but also impractical. The number of genes to be targeted should be limited since it will increase the possibility of unwanted side-effect or toxicity which may be caused by a member drug belonging to the multi-targeted drug [5]. In a word, a multi-target drug with the minimum number of target genes will be most desirable. In this regard, the gene set should play a role of blocking disease progression from onset genes to apoptotic genes. To this end, the min-cut network algorithm can be applied to a disease pathway network and it will provide a minimum target gene set. There exist many well-established implementations for the min-cut algorithm [6]. Barabási emphasized the importance of network-based approaches to human diseases in identifying new genes for complex diseases [7]. A network based computational analysis also can be used to enhance the efficiency of the drug development process. Wu et al. proposed a computational approach that finds drug targets by clustering networks through heterogeneous biomedical data that include genes, biological processes, pathways, and phenotypes [8]. Considering that the conventional means demand considerable cost and time, the approach of Wu et al. (i.e., target gene identification using available sets of biomedical data) would be an effective pre-run process ahead of proteomic analyses or in vivo tests. However, in the network of a gene set, known inflows and outflows influence the interactions between genes, and most pathway data include this kind of directional information [9]. Because such biological processes cannot be retrogressive, in silico methods should reflect these directional relations. In particular, for target gene identification, directional or causal information can be more important because the states of molecules change to innate directions and not to their opposite states. However, in the aforementioned study, the directional relations were not implemented on the network. Nevertheless, many studies have recently used directed networks that incorporate biological pathways [6, 10–13]. Chen et al. suggested a sub-pathway-based approach for analyzing drug responses, which is more computationally effective than when examining the entire pathway [10]. However, this approach is also problematic in that other genes are ignored if excluded from the subset of a pathway. Given a directed network of genes, the well-established graph algorithms can be used. By representing genes as nodes and directions as edges, various biomedical issues can be intuitively explained. To gain insights about disease progression, graph-cut algorithms can be used to identify target genes. A graph cut refers to the process of dividing nodes in a network into two groups such that no path links one group with another. Interesting studies have been conducted that use graph-cut algorithms, including for the prediction of protein functions, to address the consistency problem in multiple sequence alignment, and for hippocampus segmentation in MR images [14–16].

## Results

In this study, we propose an applied graph min-cut algorithm (Min-cut) for use with disease pathways in identifying drug-targeted genes. A cut is defined as a set of edges. The target genes we define here are those linked by these edges. A cut on the pathway network blocks the progression of a disease. Assuming that all edges have the same weight value, the minimum number of edges results in a minimum number of linked nodes. Min-cut is the minimum cut achieved with the smallest total weight of the edges. Our motivation for employing Min-cut to this study is as follows. Drug compounds can target one specific or sometimes several genes. Csermely indicated that multi-target drugs based on a network approach can help systematic drug design [17]. A graph-cut algorithm can search multiple target genes simultaneously and thus meet the requirements of drug design. However, approximately 22,000 known human genes exist, some of which may be a candidate target gene (CTG) [18, 19]. It is nearly impossible to consider all possible combinations of disease genes [20–22]. In terms of a graph cut on a pathway network, this means that every cut can provide a multiple number of CTGs. To circumvent this difficulty, we employ Min-cut to limit the number of CTGs. The proposed method is applied to 10 disease pathways including Alzheimer’s disease and type 2 diabetes mellitus. To validate the results of our experiments, we employ gene set enrichment analysis (GSEA) software and review PubMed literature and *de facto* drug targets reported in the Kyoto Encyclopedia of Genes and Genomes (KEGG) database.

### Experiment on Simulation Data

**Fig. 1:**
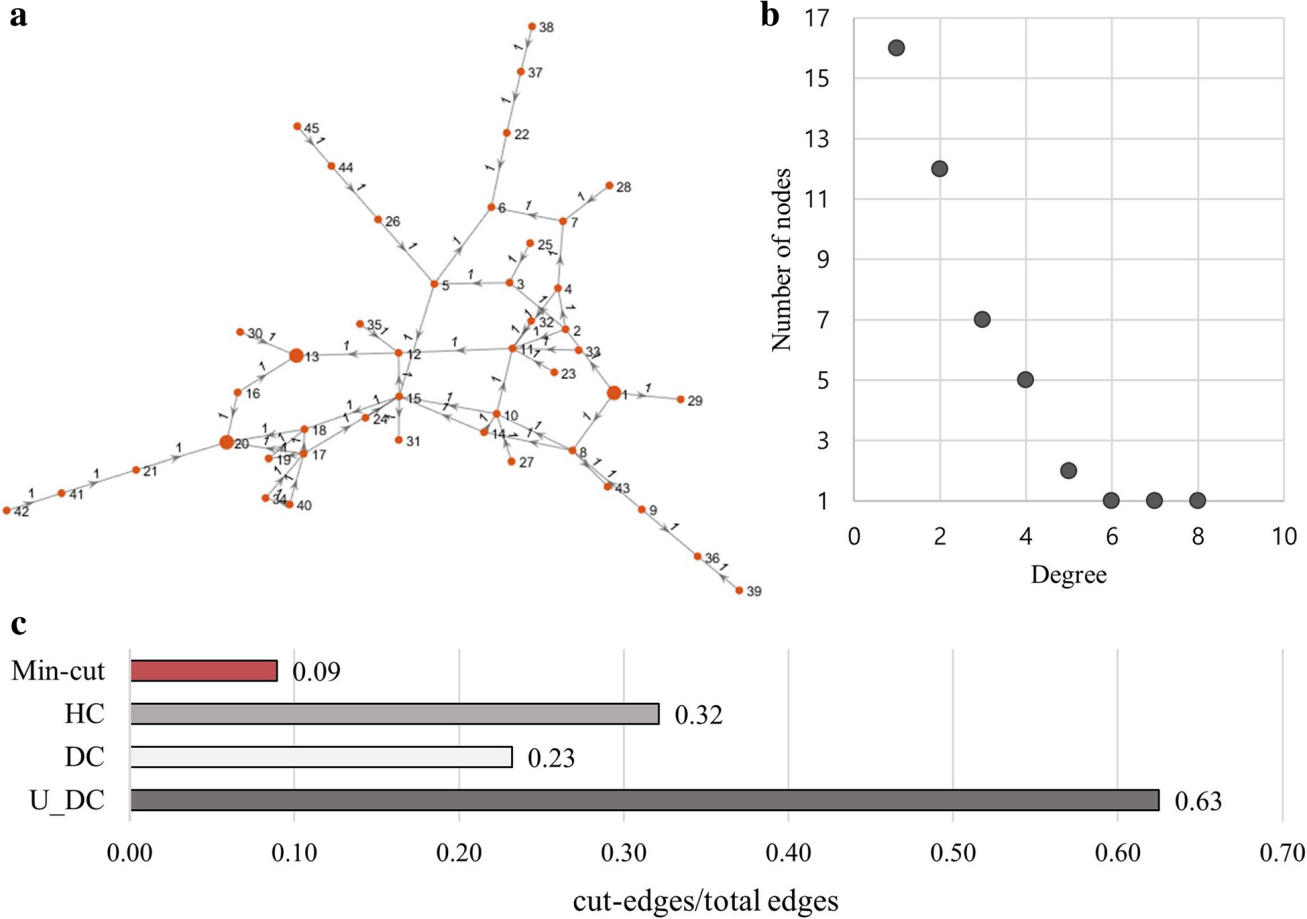
The result of the proposed method on simulated scale free network. **a** directed scale free network. **b** the plot of degree distribution. **c** result of performance comparison between the proposed Mincut based algorithm with peer methods, U_DC, DC, and HC

We applied the proposed method to the simulation data. Fig. 1(a) is the simulated network which was generated with 45 nodes and 56 directed edges and Fig. 1(b) is to plot degree distribution showing that the network is a scale free network. In order to apply Min-cut algorithm, we set source nodes (indexed 1,2,8) which have no incoming edges and sink nodes (indexed 13,20) which have no outgoing edges. There are previous target identification approaches based on network analysis. The simplest and conceptual reference is to count the degree of edges to define the most important target genes on the undirected graph, so called Undirected Degree Centrality (U_DC). And Degree Centrality (DC) is defined as the number of outgoing links incident upon a node, while U_DC includes both incoming and outgoing edges. And the Hubs Centrality (HC) are basically singular vectors of the adjacency matrix of the graph [23, 24]. The ratio of cut-edges via total edges was used as a performance measure so that the method which minimizes the edge disruption (cut edges) will be assessed as a good target gene identification method. Fig. 1(c) shows that the ratio of cut-edges from Min-cut is 0.9 and the ration of connected edges of resulting top 6 nodes from three centralities is 0.32, 0.23, and 0.63 respectively. We got a result that the Min-cut based method can suggest the minimum cut-edges by considering the disruption impact on the connection (edge) of genes, rather than the genetic changes of each node (gene).

### Experiment on Real Data

Table 1 summarizes the real data that were used to verify the proposed method: disease pathways, directed PPI, and protein-drug relations. Disease pathways were utilized to construct initial pathway networks and to set the role of genes, whether source or sink. We collected 10 disease pathways from the KEGG [25]. In order to extract unique results by setting disease specific onset or apoptotic genes, we selected one or more disease pathways involved in 6 different disease classes such as neurodegenerative diseases, metabolic diseases, and cancer, so on. (Details about disease specific genes are explained in the description of Table 2.) The KEGG database provides a manually drawn pathway map. Details of the 10 disease pathways, pathway name/ID, corresponding disease name/ID, and class of the disease, are listed in bottom of Table 1. The total number of genes involved in are 1208. For network augmentation, we employed directed PPI, which was developed by [12] to investigate intracellular signal transduction. Their resulting network includes 2626 directional relations between 1126 proteins.

**Table 1:** Data description

	Description	
Disease pathway	10 pathways of 10 diseases including 1208 genes KEGG (http://www.Genome.Jp/kegg/)	
Directed PPI	2626 directional relations between 1126 proteins (http://stke.sciencemag.org/)	
Pathway name/ID	Disease name /ID	Disease class
Alzheimer’s disease/ hsa05010	Alzheimer’s disease (AD)/H00056	Neurodegenerative diseases
Type II diabetes mellitus/ hsa04930	Type 2 diabetes mellitus (T2DM) /H00409	Endocrine, metabolic diseases
Melanoma/ hsa05218	Malignant melanoma [17]/H00038	Cancer
Prostate cancer/ hsa05215	Prostate cancer (PC)/H00024	Cancer
Amyotrophic lateral sclerosis/ hsa05014	Amyotrophic lateral sclerosis (ALS) /H00058	Neurodegenerative diseases
Huntington’s disease/ hsa05016	Huntington’s disease (HD)/H00059	Neurodegenerative diseases
Prion diseases/ hsa05020	Prion diseases (PRION)/H00061	Neurodegenerative diseases
Primary immunodeficiency/ hsa05340	Common variable immunodeficiency (CVID) /H00088	Primary immunodeficiency
Renal cell carcinoma/ hsa05211	Renal cell carcinoma (RCC)/H00021	Developmental disorder, Cancer
Nonalcoholic fatty liver disease/hsa04932	Nonalcoholic fatty liver disease (NAFLD) /H01333	Endocrine, metabolic diseases

**Table 2:** Source and sink genes

ID	Source genes	Sink genes	# of (source, sink) combination
AD	APP; CAPN1	CASP3; APBB1; MAPT	6
T2DM	INS; INSR	GLUT4	2
MEL	GF; NRAS; BRAF	CCND1; CDK4	6
PC	GF; PTEN; NKX3–1; CDKN1B	E2F1; TP53; BCL2; CASP9; BAD; FOXO1; MTOR	28
ALS	SOD1	MAP3K5; CASP3; NEFL; NEFM; NEFH	5
HD	Htt; GRM5	CASP3; ITPR1	4
PRION	PrPc	PKA	1
CVID	RAG1; RAG	ICOS	2
RCC	HGF; MET; EPAS1	SLC2A1; VEGFA; TGFB1; PDGFB; GFA	15
NAFLD	IL6; TNF; INS; LEP; ADIPOQ; FASLG	CASP3; CASP7; MAPK8	18

**Fig. 2:**
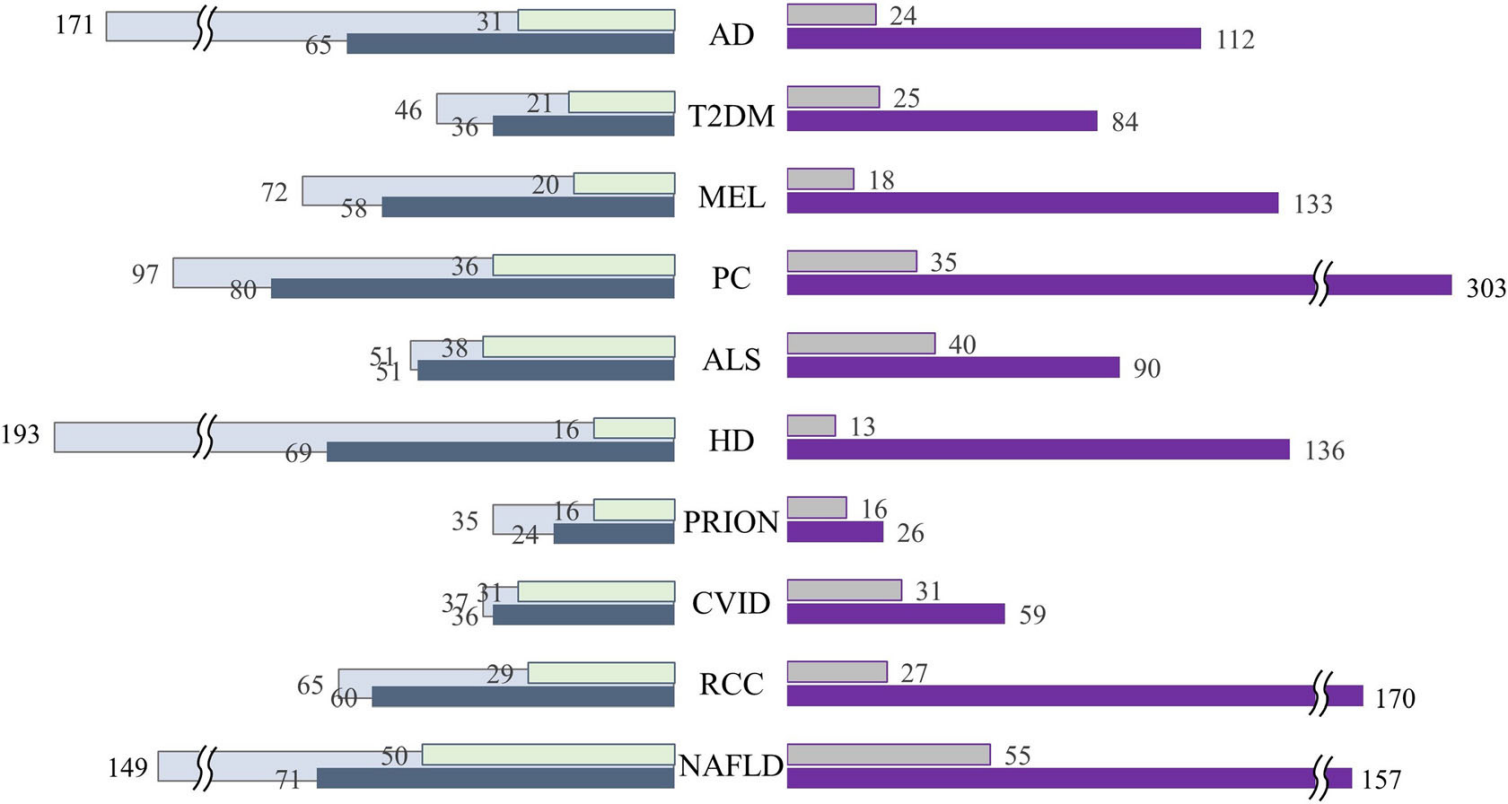
Network augmentation results

Initial pathway networks were built from each of the pathways. And the initial networks were augmented for becoming denser so that there will be not any technical problem when we apply Min-cut algorithm to the networks. First of all, we collected directional information on protein interaction network data (directed PPI) derived from the study of [12]. Then Genes that are not connected in the initial pathway are connected if their relations are indicated in the directed PPI. Therefore, edges in the initial network are augmented with edges in the directed PPI. Figure 2 shows the results of the pathway augmentation. The left side of the figure represents the toll of nodes and the other side represents the toll of edges. Bars indicating the initial network are shaded and outlined; those of the augmented network are represented with solid bars. In the case of AD, the number of connected nodes (genes) included in the network was 31 (18%) and the number of edges was 24, thus resulting in a sparse network. However, after network augmentation with directed PPI, the number of nodes and edges were increased to 210 and 467%, respectively. Across the 10 disease pathway networks, the average rate of increase in the number of nodes and edges was 207 and 454%, respectively. Not only the number of connected nodes and edges, but also additional information on the direction between nodes complemented the initial network.

Table 2 lists the source (disease onset) and sink (apoptotic) genes defined in each pathway. One or more genes per pathway were manually selected from descriptions or curated studies in KEGG. Every pair of genes (source, sink) was fed to Min-cut. For example, the number of source and sink genes for AD was two and three, respectively, and experiments were run a total of six times. This approach was similarly applied to the remaining disease pathways. The combination of (source, sink) per pathway is summarized in the last column of Table 2. Source genes tend to be specified with each disease pathway, such as APP for AD and Htt for Huntington’s disease. APP is an integral membrane protein that is expressed in many normal tissues, particularly in the synapses of neurons. Sometimes APP forms a protein basis on amyloid plaques, which are found in the brains of AD patients [26]. And the HTT gene provides instructions for making a protein called huntingtin which actives highly in the brain playing an important role in nerve cells (neurons) [27]. By contrast, sink genes such as CASP3 are commonly involved in several pathways, which thus classifies them as apoptotic genes. Apoptosis is a form of programmed cell death that occurs in multicellular organisms [28]. This means that CASP3 can be a sink gene of several diseases such as AD, HD, and NAFLD as shown in Table 2.

**Fig. 3:**
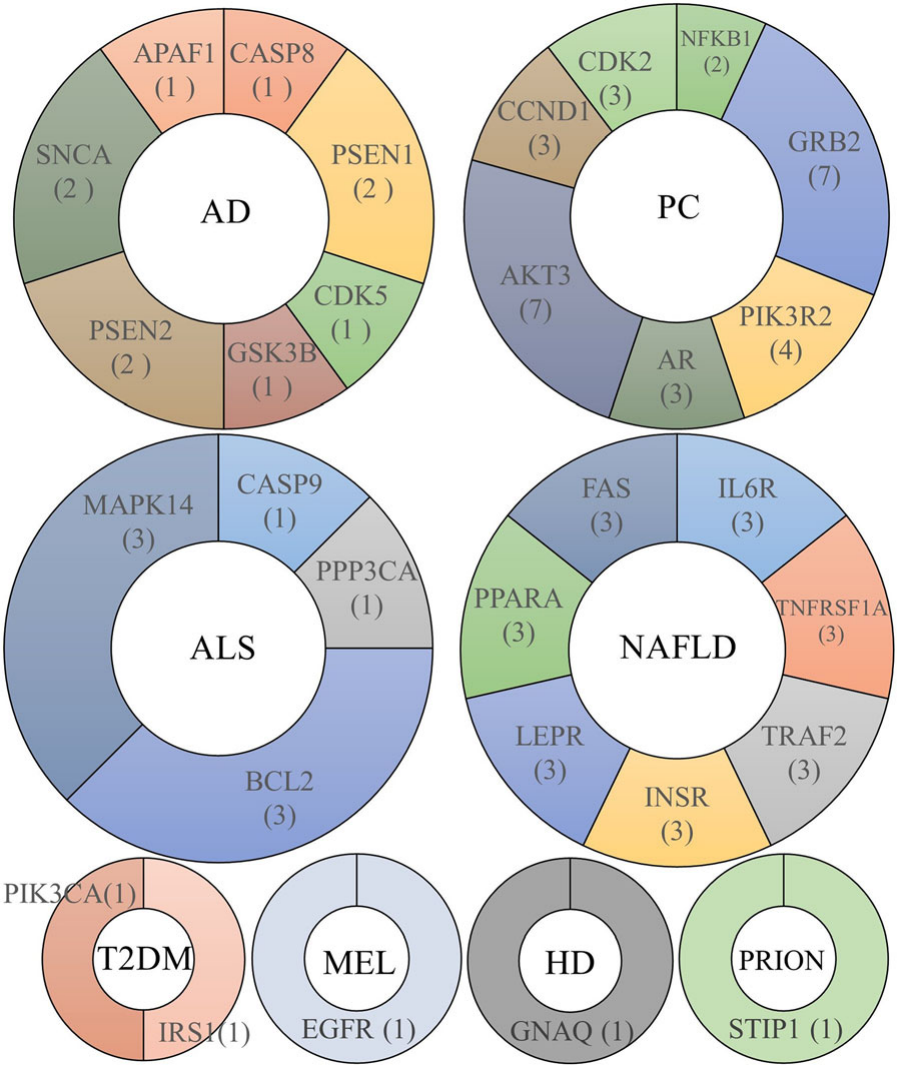
CTGs. Source and sink genes appearing in Table 2 are excluded from these charts

The pie charts in Fig. 3 show the results of CTGs identified by Min-cut. Note that the number of runs was different for each disease pathway. These different run proportions to the total number of runs should be considered. The number in parentheses indicates the number of occurrences of that gene during Min-cut runs for every combination of (source, sink) genes. The higher the occurrence rate, the more significant was the gene as a CTG. For example, in the case of AD, PSEN1 occurred twice as many times as CTG during six runs. The proportion of PSEN1 in AD was 33.3% (=2/6 × 100) The most frequently occurring CTGs in AD were PSEN1, PSEN2, and SNCA. In addition, their occurrence rates were all 33.3%. However, in the case of primary immunodeficiency (CVID) and renal cell carcinoma (RCC), we could not identify CTGs because there was no connection between source and sink genes.

**Fig. 4:**
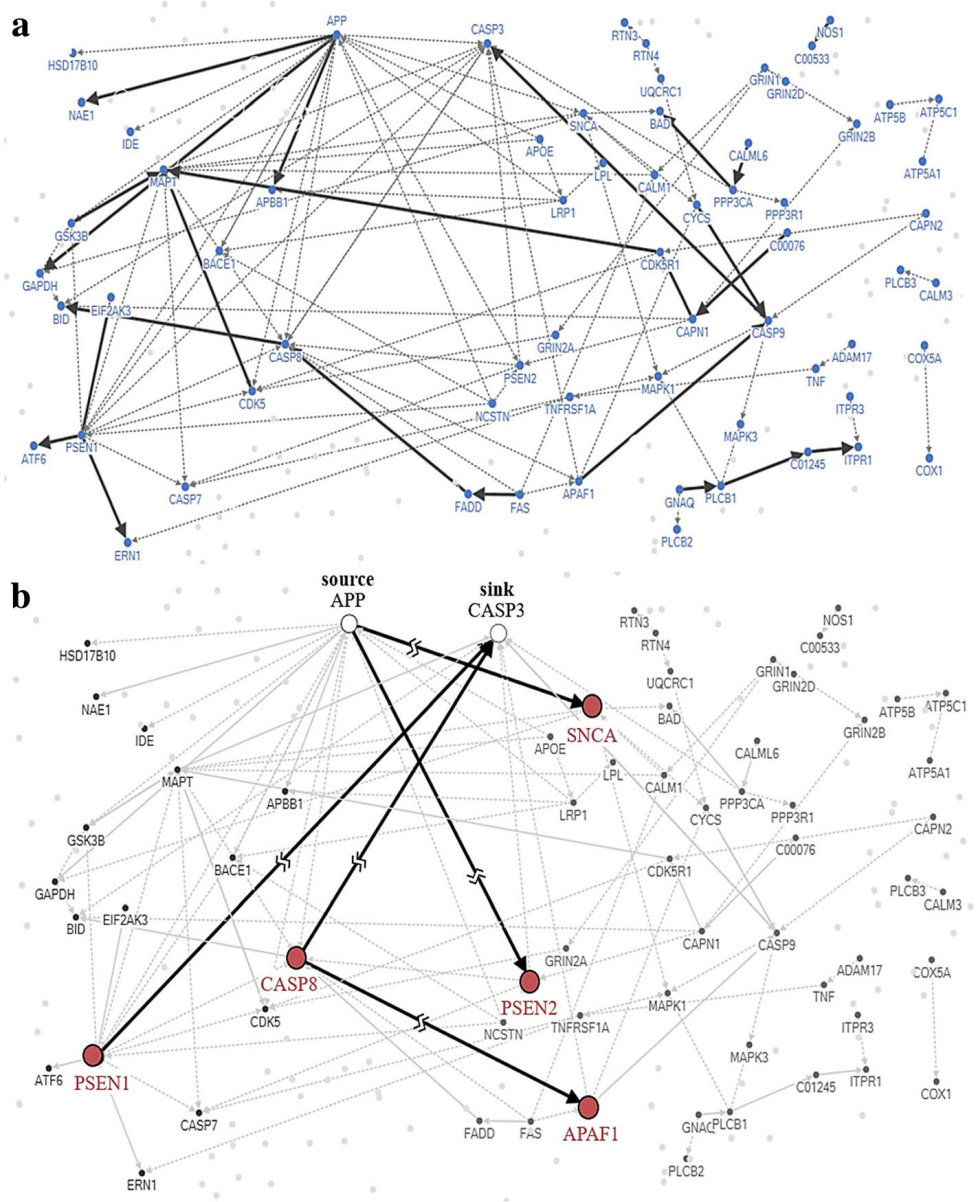
Visualization of resulting networks from Min-cut on the pathway of AD. **a** AD pathway network constructed with gene-gene interactions in the AD pathway (solid line) and directed PPI (dotted line). **b** Results of CTGs by Min-cut

**Fig. 5:**
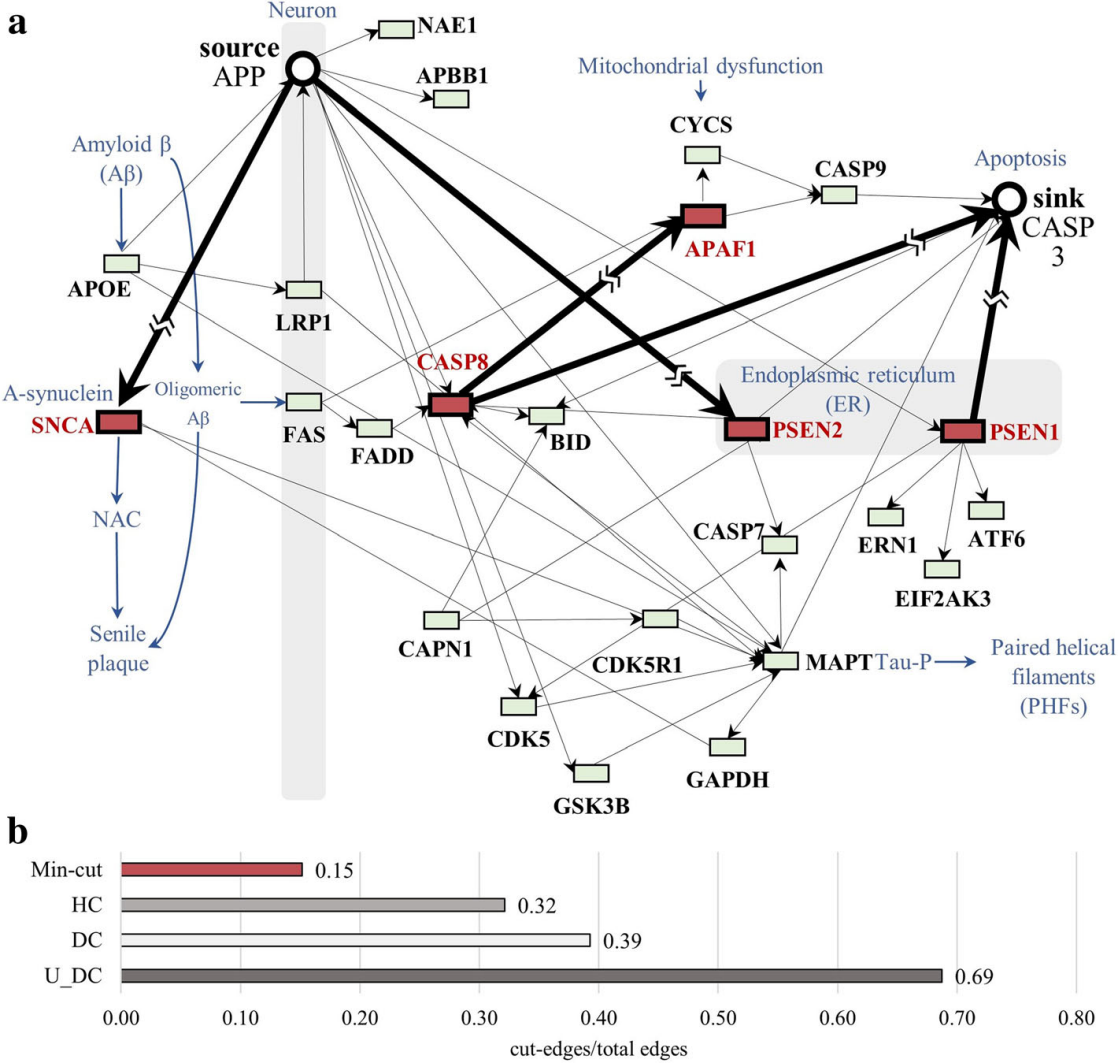
**a** Illustration for cut-edges and the CTGs in AD pathway from KEGG. **b** comparison of the resulting CTGs on AD with previous network-based essential gene identification methods, Degree Centrality and Hubs method for AD and ALS

As a representative example, Fig. 4 shows the resulting networks of Alzheimer CTGs with source and sink genes. Solid edges in Fig. 4(a) are from pathway data of 24 relations between 31 genes of 171 total disease-related genes (connected nodes = 18%). The dotted edges indicate directed PPI. In this example, 112 relations between 65 genes were augmented. Figure 4(b) shows the results of Min-cut applied to the augmented network. In the figure, APP and CASP3 were set as a pair of (source, sink). Min-cut successfully disconnected the network with minimal effort; five edges were cut. Along the respective edges, five CTGs were identified: PSEN1, CASP8, SNCA, PSEN2, and APAF1A. Those are marked with solid circles. And Fig. 5(a) is Illustration for cut-edges and the CTGs in AD pathway from KEGG an illustration for cut-edges and CTGs in the AD process sourced from KEGG pathway. As it shows, SNCA plays an important role changing amyloid beta to the senile plaques which are extracellular deposits of amyloid beta in the grey matter of the brain. Fig. 5(b) shows that the ratio of cut-edges from Min-cut is 0.15 and the ration of connected edges of resulting top 7 nodes from three centralities is 0.32, 0.39, and 0.69 respectively.

**Fig. 6:**
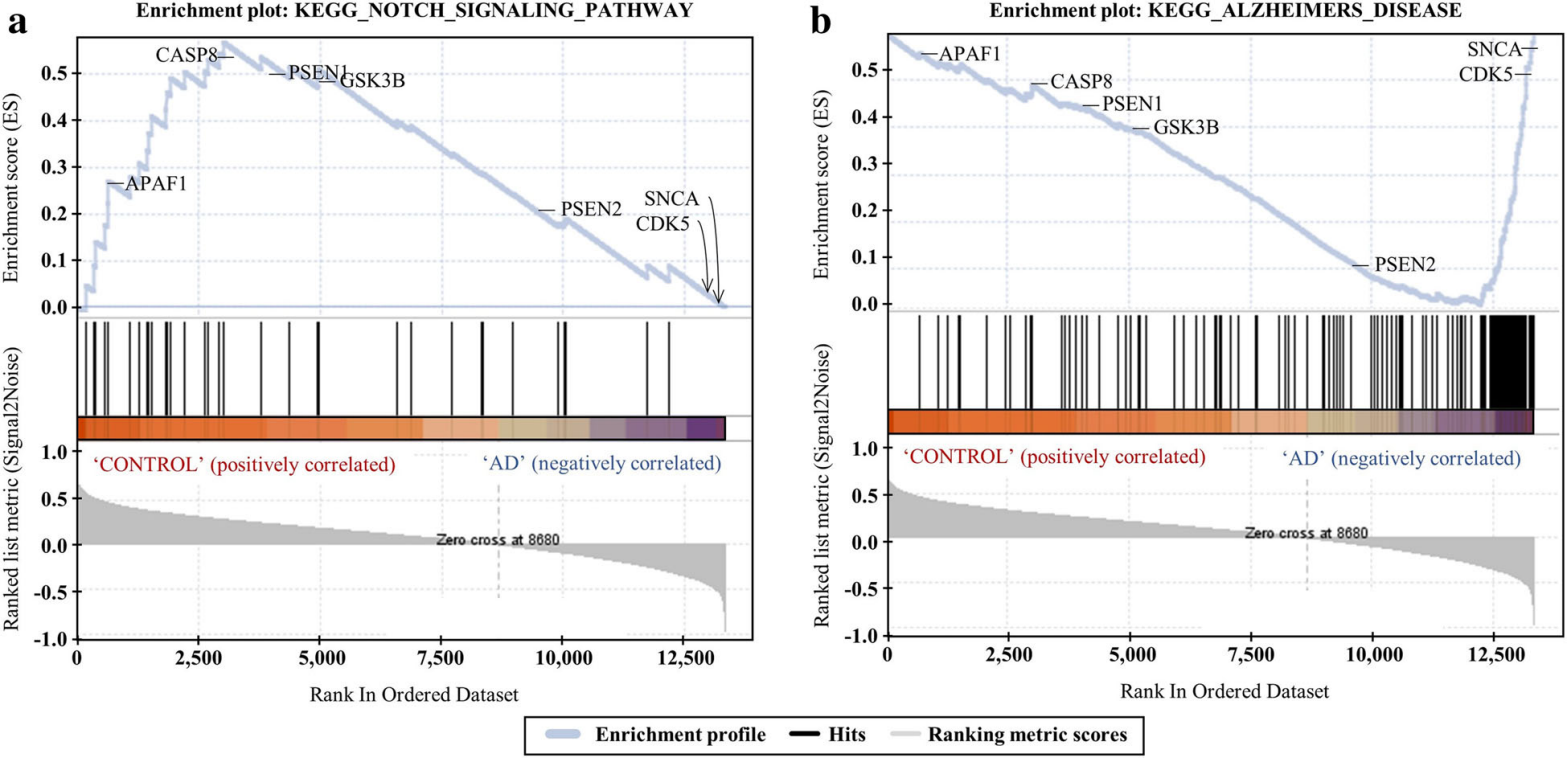
Gene set enrichment analysis: **a** Control: KEGG notch signaling pathway. **b** AD: KEGG Alzheimer’s disease pathway

To verify CTGs identified in our experiments, we conducted GSEA, and reviewed PubMed literature and *de facto* drug targets reported in the KEGG database. In this study, we provide validation results for AD. Figure 5. shows a comparison of the two sets (AD and control). We found that most genes involved in the KEGG AD pathway were DEGs of the AD phenotype in GSEA. This indicates that our obtained initial network from the AD pathway is a reasonable and an efficient means to find markers. Each of the AD CTGs that we identified are shown in the different panels in Fig. 6. where The upper panel (a) shows ES patterns for the control: a KEGG notch signaling pathway containing 34 genes. The overall profile of the result indicates that ES is positively correlated with a phenotype, the maximum deviation of ESs from zero reaches 0.567, and the nominal *p* value is 0.010. By contrast, the lower panel (b) shows ES patterns for AD: a KEGG Alzheimer’s disease pathway containing 140 genes. The overall profile of the AD result indicates that ES is negatively correlated with a phenotype, the maximum deviation of ESs from zero reaches − 0.576, and the nominal p value is 0.008. The two patterns are significantly different, and a sudden increase in ES in the lower panel provides evidence that the genes in the AD pathway are significant. In our AD CTGs, four genes are located at the rightmost side of the graph, and thus appear to be target genes: SNCA (ES: -0.415), CDK5 (ES: -0.402), CDK5R1 (ES: -0.320), and PSEN1 (ES: -0.094).

The following are typical findings from previous studies on CTGs of AD. The CTGs of AD in Fig. 3 were derived from PubMed literature. More results are provided in Table 3. ***SNCA***: Non-Aβ component of AD amyloid precursor SNCA gene may contribute to an increased risk of AD. SNCA gene polymorphism may be associated with an increased risk of AD [29, 30]. ***CDK5***: Cyclin-dependent kinase 5 is reported to intimately associate with the process of the pathogenesis of AD. CDK5/CDK5R1 protein kinases involved in abnormal tau phosphorylation in AD. Tau proteins are widely known to be associated with dementias of the nervous system such as Alzheimer’s disease and Parkinson’s disease [31–33]. ***PSEN1***: Mutations in the presenilin 1 gene are the most frequent cause of familial AD. There are reports about PSEN1 mutations in various species including Turkish, Chinese, and Korean [34–36].

**Table 3:** The list of validation results on PubMed literatures

Disease name	Candidate Target Genes	PMID
AD	PSEN1	24927704, 24718101, 24928006, 25045597, 24416243, 20388456, 21501661, 25595498, 22503161, 18437002, 24906965, 22618995
	CASP8	28985224
	CDK5	28714390, 23816988
	GSK3B	24101602, 25420549, 20576277, 18932008, 18852354, 17028556
	PSEN2	24927704, 25104557, 25045597, 24838203, 26203236, 20164579
	SNCA	24777780, 27567856, 27184464, 18322368
	APAF1	–
	CDK5R1	21130128
T2DM	IRS1	24612564, 21917432, 24584551, 21834909, 19734900, 14633864
	PIK3CA	28934129, 28477532
MEL	MAP2K1	28881731, 23174022, 22197931
	MAPK1	24468268, 24158781
	EGFR	29311018, 29121185
	PIK3R2	–
	ARAF	24962318
	PIK3CA	28972077, 26343386
PC	AR	29460922; 29464071; 29462692;
	EGFR	–
	GRB2	25153383;
	PIK3R2	26677064;
	NFKB1	–
	AKT3	25153383; 28624527; 28150530;
	CCND1	29142597;
	CDK2	29323532; 27819669;
AL	CASP9	–
	PPP3CA	–
	BCL2	24737943, 21678416, 21624464
	MAPK14	–
	C16844	–
HD	GNAQ	–
PRION	STIP1	–
NAFLD	IL6R;	–
	TNFRSF1A;	–
	TRAF2;	–
	INSR;	29325294, 29254185
	LEPR;	27470889, 27257426, 26965314
	PPARA;	29327584; 28077274;
	FAS	29345914;

Among the CTGs we discovered and as shown in Fig. 3 are * de facto* drug targets. The following target genes and drugs are also listed in Table 4: PSEM1 for Alzheimer’s disease, INSR for Type II diabetes mellitus, MAP2K for Melanoma, and AR for prostate cancer. These have been already developed as drugs to treat the diseases in practice. The rest of our CTGs also have potential to be biomarkers as drug targets.

**Table 4:** Validation of *de facto* drug targets

ID	Target proteins	Drug
AD	PSEN1 (HSA:5663)	Begacestat (D08869) /Tarenflurbil (D09010) /Semagacestat (D09377) /Avagacestat (D09869)
T2DM	INSR (HSA:3643)	Insulin (D00085) / etc. 19 insulin related drugs
MEL	MAP2K (HSA:5604)	Cobimetinib (D10405) /Cobimetinib fumarate (D10615)
PC	AR (HSA:367)	Testosterone (D00075) /Flutamide (D00586) /Bicalutamide (D00961) /Nilutamide (D00965) /Enzalutamide (D10218)

## Discussion

Our study is based on the notion that target genes interrupt the progress of a disease. The resulting CTGs of our Min-cut are points at which the flow from disease onset genes to apoptotic genes can be cut. The visualized CTGs on the pathway network can help in understanding the mechanisms involved in disease progression and the roles that CTGs play therein. And the proposed method offers new insights into disease treatment and drug development. The CTGs can be recommended as preferential subjects to improve the treatment of diseases and drug design. Although CTGs have not been fully validated, we believe that they have the potential to be primary drug targets from of a considerable number of genes.

## Conclusions

In this study, we proposed the pathway Min-cut algorithm for target gene identification. It is assumed that if the network of a disease pathway is disconnected, development of the disease will not continue. To find points along the pathway that can be “cut,” while performing this task at a minimal cost, Min-cut algorithm was developed. We then applied it to a network augmented with additional information on gene-gene relations, including the causalities between them. Given source and sink genes, the proposed algorithm found an edge set that blocks every flow from a source to a sink gene. The candidate genes were validated through diverse means, namely, gene expression profiling by GSEA, the findings from various studies, and existing drugs. This work can be complemented if the biological domain produced a greater number of novel discoveries in areas such as gene-gene relations, disease pathways, gene expression and mutation, and so on.

## Methods

**Fig. 7:**
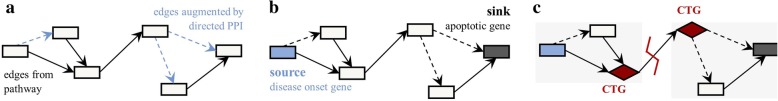
Proposed Method: **a** Disease pathway network augmentation with directed PPI, **b** source and sink genes, (**c**) Min-cut for candidate target gene identification

Figure 7 illustrates the overall procedure of our study. First, a network is composed of a disease pathway. Each node indicates disease related genes and a directed edge between two different genes indicates that one gene may have biological changes in that direction. And then the initial network augmented with directed PPI information to endow causality on flows on the network, as shown in Fig. 7(a) [12]. Solid black edges are from pathway data and dotted blue edges are from directed PPI data. Second, as shown in Fig. 7(b), source and sink genes are chosen, where source genes may be considered responsible for the onset of a disease and sink genes may lead to apoptosis. One or more genes per pathway were manually selected from descriptions or curated studies in KEGG. Every pair of genes (source, sink) was fed to Min-cut. Finally, as shown in Fig. 7(c), Min-cut finds the smallest sum of edges necessary to disconnect (i.e., “cut” the pathway of) a disease-onset gene and an apoptotic gene [37, 38]. The resulting multi-genes, which are linked by the cut edges, are identified as CTGs. Details of the method are provided in the following subsections.

### Disease pathway network and augmentation

In our method, each disease pathway is represented as a network *G* = (*V*, *E*) that consists of genes as nodes *V* and relations between genes as edges *E*. In this initial network, a significant number of nodes are not connected. Therefore, the network is augmented with biological domain knowledge and is endowed with causality on its edges. There are some technical benefits to this network augmentation. First, the network becomes denser; if the network is sparse, applying Min-cut is difficult. Second, directionality reduces the solution space by eliminating unnecessary paths from the network. The directional information on protein interaction network data (directed PPI) is derived from the study of [12]. Genes that are not connected in the initial pathway are connected if their relations are indicated in the directed PPI, as shown in Fig. 7 (a). Therefore, edges in the initial network *E* are augmented with edges in the directed PPI$\overrightarrow{E}$.

### Source and sink genes

In the augmented network, defining sets of source nodes *S* and sink nodes *T*, as shown in Fig. 7(b), is required. In the case of source nodes, some genes can be found in the KEGG description or the well-known study in PubMed. They tend to be located at the beginning of the pathway because the pathway describes sequential changes of state from normal to abnormal. Although the source genes have a normal status, they may cause the disease. For example, amyloid precursor protein (APP), which appears at the beginning of the Alzheimer’s disease (AD) pathway, can be defined as a source node *s* ∈ *S*. However, sink genes are generally found at the end of the pathway in which apoptosis or a disorder status are described. In several pathways including the AD pathway, CASP3 can be defined as a sink node *t* ∈ *T*. This protein is a member of the cysteine-aspartic acid protease (caspase) family. Sequential activation of caspases plays a central role in the execution-phase of cell apoptosis.

### Pathway partitioning using min-cut

**Fig. 8:**
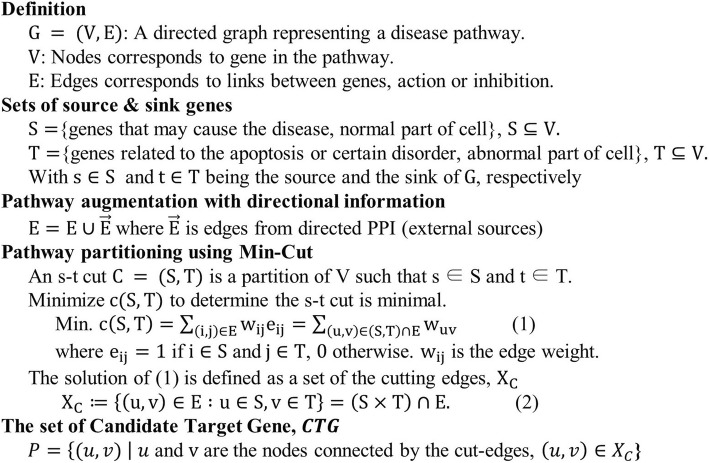
Pseudo-code for pathway Min-cut

The proposed method employs a Min-cut algorithm [37, 38] to find CTGs. We assume that cutting an edge with direction from one gene to the other means that blocking the change from one gene to the other. That is, we define genes connected to the cut edges as CTGs. The ultimate goal of the algorithm is to find a set of genes connected to the cut edges. The number of edges cut by Min-cut can be multiple if multiple paths exist from the source to the sink. The algorithm minimizes the number of cut edges, technically to improve efficiency of the marker development process and biologically to avoid unwanted side effects by selecting too many genes together. In this process, the genes connected to the cut edges become candidate target genes because the multiple cut edges completely disconnect the onset gene (source) from the apoptotic gene (sink). For example, when there are two nodes (genes) A, B and a connected directional edge (AB) from A to B, cutting (AB) indicates biologically blocking gene A to be transformed to gene B. This is what we traditionally try to do in the targeted treatment and drug development. In short, cutting a certain edge refers to developing treatments by targeting two genes of the cut-edge. Once source *S* and sink nodes *T* are determined in the network, Min-cut finds the edges minimizing  the following functional:$\operatorname{Minimize}\ \mathrm{c}\left(S,T\right)={\sum}_{\left(i,j\right)\in E}{w}_{ij}{e}_{ij}={\sum}_{\left(u,v\right)\in \left(S,T\right)\cap E}{w}_{uv}$where *c*(*S*, *T*) denotes the *s-t* cut capacity, which is the sum of edge weights, *w*_*ij*_*e*_*ij*_. The value of *w*_*ij*_ is large if the connection is strong, and vice versa. In addition, *e*_*ij*_ is 1 if nodes *i* and *j* are connected, and 0 otherwise. Note that edges in the pathway network are not weighted, and thus *e*_*ij*_ is ignored. Regarding source and sink genes, the capacity becomes the sum of *w*_*uv*_**,** where (*u*, *v*) ∈ (*S*, *T*)***.*** In our pathway application, the cut edges found by Min-cut may be regarded as the border of disease progression from normal to abnormal status. Then, it is assumed that the genes connected by the cut edge become a set of CTGs. Figure 6(c) illustrates the idea of cutting edges at the minimum capacity. Figure 8 provides the pseudo-code giving further details.

### Gene set enrichment analysis

We interpreted the resulting CTGs by profiling gene expression. GSEA is a computational method that indicates whether predefined gene sets (pathway) reveal statistically significant, considering the two phenotypes [39, 40]. Much research has been conducted based on the assumption that differentially expressed genes (DEGs) may be potential biomarkers [41–43]. In case of the AD, a gene expression dataset (GSE1297) was obtained from GEO that contains 13,321 gene expression values for two classes, one for AD and the other for a control. GSEA provides a ranked list that is based on the gene differential expression between the classes for the entire range of genes. More importantly, an enrichment score (ES) is calculated by moving down the ranked list and increasing a running-sum statistic whenever a gene in a set is encountered, while decreasing it when genes are not in an a priori defined set of genes such as a pathway. This will then reflect the degree to which a set is overrepresented at the extremes (top or bottom) of the entire ranked list. For details on GSEA, see the study of [39].

## Abbreviations

AD: Alzheimer’s Disease
ALS: Amyotrophic lateral sclerosis
APP: Amyloid precursor protein
CTG: Candidate Target Gene
CVID: Common variable immunodeficiency
DC: Degree Centrality
DEGs: Differentially expressed genes
directed PPI: directed protein-protein interaction
ES: Enrichment score
GEO: Gene Expression Omnibus
GSEA: Gene Set Enrichment Analysis
HD: Huntington’s disease
KEGG: Kyoto Encyclopedia of Genes and Genomes
MEL: Malignant melanoma
NAFLD: Nonalcoholic fatty liver disease
PC: Prostate cancer
PRION: Prion diseases
RCC: Renal cell carcinoma
T2DM: Type 2 diabetes mellitus
